# Trends of Stroke Incidence and 28-Day All-Cause Mortality after a Stroke in Malaysia: A Linkage of National Data Sources

**DOI:** 10.5334/gh.791

**Published:** 2021-05-26

**Authors:** Wen Yea Hwong, Swee Hung Ang, Michiel L. Bots, Sheamini Sivasampu, Sharmini Selvarajah, Wan Chung Law, Lydia Abdul Latif, Ilonca Vaartjes

**Affiliations:** 1 Institute for Clinical Research, National Institutes of Health, Ministry of Health, Selangor, MY; 2 Julius Center for Health Sciences and Primary Care, University Medical Center Utrecht, Utrecht University, NL; 3 Sharmini Selvarajah Consulting, Selangor, MY; 4 Neurology Unit, Department of Medicine, Sarawak General Hospital, Ministry of Health, Kuching, MY; 5 Regen Rehab Hospital, Petaling Jaya, MY

**Keywords:** stroke, incidence, mortality, developing countries, Malaysia

## Abstract

**Background:**

Data on nationwide trends for stroke metrics are crucial to understand the extent of the disease burden to a country’s health system. Yet, this information remains scarce in low- and middle-income countries.

**Objectives:**

This study investigated trends of stroke incidence and 28-day all-cause mortality after a stroke from 2008 to 2016 in Malaysia, through linkage across national data sources.

**Methods:**

Hospital admissions with a principal diagnosis of stroke or transient ischemic attack were included. Cases with first stroke were identified through linkage of hospital admission registers where age and sex-standardized trends of stroke incidence and its subtypes were calculated. By linking hospital registers to the National Death Register, the 28-day all-cause mortality rates after a stroke were estimated. Mann-Kendall’s test was used for trend evaluation.

**Results:**

From 243,765 records, the trend of stroke incidence showed an increase of 4.9% in men and a drop of 3.8% among women. Incidences were higher in men, at 99.1 per 100,000 population in 2008 and 103.9 per 100,000 in 2016 than women (80.3 per 100,000 in 2008 and 77.2 per 100,000 in 2016). There was a substantial increase in stroke incidence among those below 65 years old, with the largest increase of 53.3% in men aged between 35–39 years and 50.4% in women of similar age group. The trend for 28-day all-cause mortality showed a decline for men at –13.1% and women, –10.6%. Women had higher mortality from stroke (22.0% in 2008 and 19.7% in 2016) than men (19.4% in 2008 to 17.2% in 2016).

**Conclusion:**

This first empirical study on stroke trends in Malaysia revealed a worrying increase in stroke incidence among the younger population. Despite a declining trend, mortality rates remained moderately high especially in women. Comprehensive strategies to strengthen the prevention and management of stroke care are warranted.

## Introduction

Despite being known as a disease of affluence, the incidence of stroke in low and middle-income countries (LMICs) has superseded that of the high-income countries (HICs) by 20% between 2000 and 2008. An increase in stroke incidence among young adults between 20 and 64 years has also been observed with a large proportion attributable to LMICs [1]. As for mortality, although both regions showed downward trends between 1985 to 2005, estimates of one-month mortality from stroke in LMICs were much higher than HICs [2].

Malaysia is a country with an emerging economy in Southeast Asia where stroke is increasingly being recognized as a major public health concern. With stroke being estimated as the third leading cause of death in the country [3], efforts to measure important local stroke metrics have been attempted. While modelled estimates are available within the Global Burden of Disease Study [4], empirical stroke incidence was first estimated in a district of Penang Island with an age-standardized rate of 67 per 100,000 population between 2010 and 2011 [5]. Short-term mortality after a stroke event in Malaysia varied from 7.8% to 34% [6,7,8]. Most of these studies however, are single-centred with cross-sectional designs. Conduct of longitudinal cohorts are scarce because they are expensive and time consuming. Establishing reliable trends for stroke metrics across time in cohorts that are more nationally representative are therefore, still lacking.

One promising strategy to possibly resolve this, is research using data linkage. With this method, information for a specific patient can be linked from multiple readily available data sources. Research using data linkage has been shown to not only influence health policy and improve preventive health strategies but more importantly, it provides an opportunity to monitor disease burden and patient outcomes in a real-time manner [9].

Thus, the aims of this study were to estimate trends of stroke incidence and 28-day all-cause mortality after a stroke across Ministry of Health hospitals in Malaysia from 2008 to 2016, via linkage of national data sources.

## Methods

### Data source

Data were sourced from two databases: 1) hospital admission database (Sistem Maklumat Rawatan Pesakit (SMRP)) within the Health Information Management System and 2) National Death Register. In brief, the SMRP is a centralized database of the Ministry of Health that collects hospital admission records from Ministry of Health hospitals since 1981, with availability of granular data from 1999. It has expanded to include private hospitals in 2008 as well as university and Ministry of Defence hospitals in 2017. For this study, only data from the Ministry of Health’s hospitals were included to avoid inconsistency in terms of data availability. While initially all documentation were entered manually, a web-based online system was subsequently started in 2012.

The National Death Register is a central registration for all deaths in the country. Death records are stored within a computerized registration system (National Population Registration System (SPPN)) [10].

### Population selection

**Figure 1:**
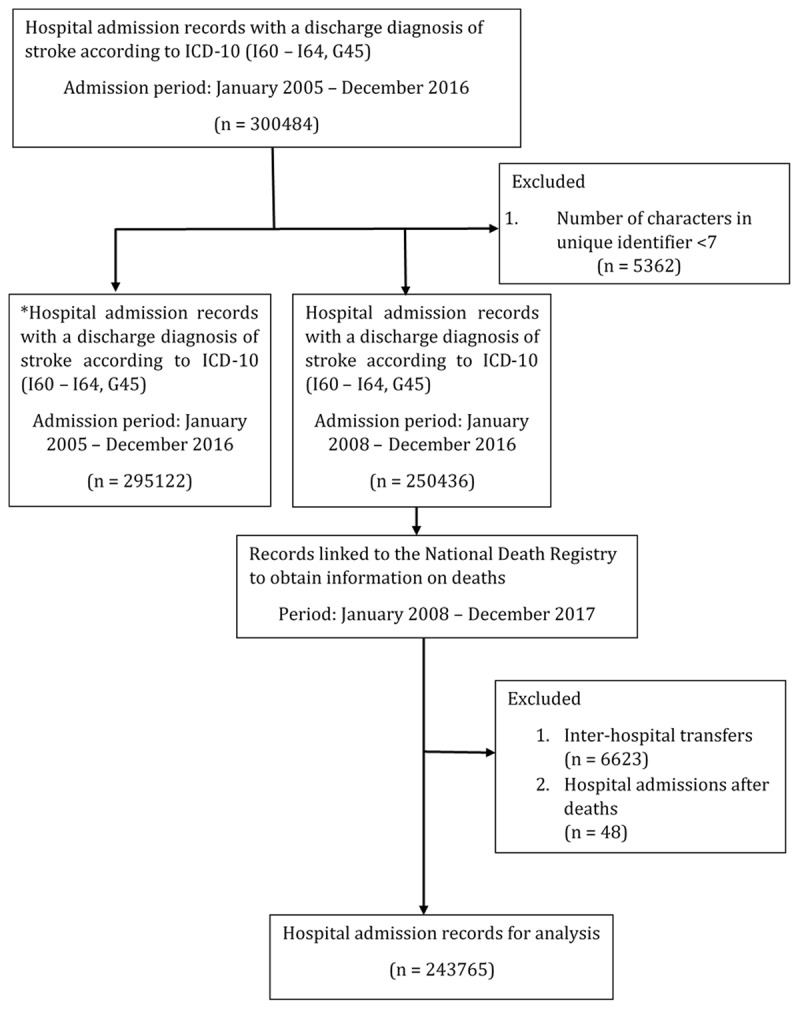
**Flow chart showing the process of data linkage and patient selection for analysis.** * Dataset used to identify patients with previous hospital admission for stroke in the last 3 consecutive years.

We included all admissions registered with a principal diagnosis of stroke or transient ischemic attack (TIA) at discharge between January 2005 and December 2016. Records were excluded for inter-hospital transfers for the same stroke event, hospital admissions after recorded deaths for the same identifier, and records with missing or inadequate unique identifiers. In Malaysia, unique identifiers that are provided for hospital admissions include either 1) the national identification number (MyKad) which was introduced in 1990, 2) an older version of the national identification number, 3) police identification number or 4) passport number for foreigners. As the least number of characters for these identifications is seven, about 1.7% records with inadequate unique identifiers were removed to improve the accuracy of the linkage. Figure 1 illustrates a flow chart on the process of inclusion and exclusion of the records.

A diagnosis of stroke or a TIA is based on clinical assessment and confirmed on imaging using either a computed tomographic or magnetic resonance imaging. The updated universal definition for stroke is published in 2013 [11]. In 2009, the expert committee has proposed to define TIA as ‘a transient episode of neurological dysfunction caused by focal brain, spinal cord, or retinal ischemia without acute infarction [12].’ In other words, neurological dysfunction that resolves within 24 hours with normal brain imaging is considered as episodes of TIA.

Diagnoses at discharge are coded to the International Classification of Disease-10 codes. Consistent with definitions from the coding, stroke subtypes in this study is defined as I63–I64 and G45 for ischemic stroke/TIA, I61 – I62 for hemorrhagic stroke, and I60 for subarachnoid haemorrhage.

### Cohort identification

Two age- and sex-specific components of stroke metrics and its subtypes were estimated in this study: 1) incidence of stroke and 2) 28-day all-cause mortality after a stroke between 2008 and 2016.

#### 1) Incidence

Incidence of stroke in this study is defined as the number of people with first-ever hospitalized stroke. It was not possible to identify whether the stroke episode was a first or a recurrent event from the admission records. For every hospital admission, a new hospital record will be added and thus, a person can be entered multiple times into the database. To overcome this issue, we created a random identification number (RIN) by combining one unique identifier and two other non-unique identifiers which were sex and date of birth with the aim to uniquely identify a patient. Use of more than one variable for linkage purposes has been associated with an improvement in the accuracy to identify patients [13].

From there, a cohort of hospitalized stroke patients was constructed by identifying the first hospitalized stroke admission every year for each RIN. Through linkage with previous hospital admission records, we excluded patients who had previous admissions for stroke within the last three consecutive years. We repeated this exercise for every year between 2008 and 2016 to obtain yearly incident cases of stroke. This similar method has been used previously in other countries for estimation of incidence using data from hospital admission records [14].

#### 2) The 28-day all-cause mortality

The 28-day all-cause mortality includes hospitalized stroke patients who died from any cause within 28 days after their stroke events. Using that as a basis, we linked the admission records for stroke to the National Death Register between year 2008 and 2017 to obtain information on date of deaths. For patients with multiple stroke admissions, only their latest stroke episode was included. Similarly, exact matching for data linkage between two databases was performed using the created RIN.

### Ethical considerations

Ethics approval was obtained from Medical Research and Ethics Committee, Ministry of Health Malaysia (ID: NMRR 18-100-39847). A waiver to obtain informed consent from patients was granted as this study utilized routinely collected data from administrative databases.

### Statistical analysis

Firstly, the number of incident cases was calculated per 100,000 Malaysian population by age and sex before being age-standardized to the 2016 Malaysian population as well as the standard world population [15]. The population size at midpoint of the year obtained from the Department of Statistics Malaysia were used. Scatter plots were presented to visualize changes for stroke incidence between 2008 and 2016. The extent of change between the years were calculated with the formula below:

{\rm Percentage\ of\ change\ in\ rate}\, = \,\frac{{\left({{\rm Rate\ in}\ 2016 - {\rm rate\ in}\ 2008} \right)}}{{{\rm Rate\ in}\ 2008}} \times \,100\%

In addition, statistical significance of the trends was estimated with the Mann-Kendall’s trend test.

Secondly, the estimates for 28-day all-cause mortality were based on the number of hospitalized stroke patients who died within 28 days after their stroke events out of total hospital admissions for stroke within a particular year. This metrics was then presented in proportions and age-standardized to the age structure for 2016 total hospital admission for stroke to assess trends. Similar analyses to incidence rates were performed, which includes scatter plots, percentage of change in mortality across the years and Mann-Kendall’s trend test.

A p-value of p < 0.05 is taken for statistical significance. Data were analyzed with R Studio 1.1.463 [16].

## Results

### Overall stroke

Table 1 shows baseline characteristics of hospital admissions for stroke from year 2008 to 2016. Apart from a drop in 2012 and 2013, the number of patients who were admitted for stroke steadily increased. Over the 9 years, there were moderate shifts in age groups of the highest proportion of hospitalized stroke patients. A downward trend in the proportion of stroke patients between the ages of 65 to 74 years was observed. In contrast, the proportion of stroke patients increased for younger patients aged 35 to 44 years and 55 to 64 years. No large differences were observed between men and women.

**Table 1:** Baseline characteristics of hospital admissions for stroke from 2008 to 2016.

	Year																	

	2008(n)		2009(n)		2010(n)		2011(n)		2012(n)		2013(n)		2014(n)		2015(n)		2016(n)	

Total admissions for stroke	24197		25351		26539		29992		20544		26537		30815		32749		33664	
Hospital transfers	534		638		675		911		594		734		850		827		860	
Hospital admissions for stroke (excluding transfers)	23663		24713		25864		29081		19950		25803		29965		31922		32804	
**Baseline characteristics**	**n**	**(%)**	**n**	**(%)**	**n**	**(%)**	**n**	**(%)**	**n**	**(%)**	**n**	**(%)**	**n**	**(%)**	**n**	**(%)**	**n**	**(%)**

Mean age (SD)	61	(16)	61	(16)	61	(16)	61	(16)	61	(15)	61	(15)	61	(15)	61	(15)	61	(15)
Age groups																		
0–34	1287	5.4	1482	6.0	1404	5.4	1555	5.3	967	4.8	1262	4.9	1597	5.3	1582	5.0	1648	5.0
35–39	561	2.4	628	2.5	676	2.6	709	2.4	519	2.6	658	2.6	916	3.1	963	3.0	1030	3.1
40–44	1018	4.3	1087	4.4	1144	4.4	1353	4.7	871	4.4	1159	4.5	1342	4.5	1553	4.9	1661	5.1
45–49	1697	7.2	1790	7.2	1851	7.2	2070	7.1	1472	7.4	1971	7.6	2268	7.6	2364	7.4	2500	7.6
50–54	2459	10.4	2537	10.3	2727	10.5	3105	10.7	2169	10.9	2701	10.5	3233	10.8	3374	10.6	3519	10.7
55–59	2733	11.5	2925	11.8	3226	12.5	3537	12.2	2476	12.4	3162	12.3	3764	12.6	4009	12.6	4184	12.8
60–64	3025	12.8	3081	12.5	3364	13.0	3862	13.3	2614	13.1	3383	13.1	3947	13.2	4226	13.2	4413	13.5
65–69	3335	14.1	3279	13.3	3302	12.8	3559	12.2	2584	13.0	3281	12.7	3894	13.0	4198	13.2	4225	12.9
70–74	3169	13.4	3337	13.5	3384	13.1	3719	12.8	2430	12.2	3197	12.4	3427	11.4	3538	11.1	3530	10.8
75–79	2237	9.5	2289	9.3	2380	9.2	2809	9.7	2040	10.2	2608	10.1	2970	9.9	3200	10.0	3075	9.4
80–84	1341	5.7	1378	5.6	1502	5.8	1720	5.9	1131	5.7	1438	5.6	1620	5.4	1757	5.5	1849	5.6
85+	801	3.4	900	3.6	904	3.5	1083	3.7	677	3.4	983	3.8	987	3.3	1158	3.6	1170	3.6
Sex																		
Men	13568	57.3	14343	58.0	14841	57.4	16901	58.1	11616	58.2	15049	58.3	17708	59.1	18878	59.1	19385	59.1
Women	10095	42.7	10370	42.0	11023	42.6	12180	41.9	8334	41.8	10754	41.7	12257	40.9	13044	40.9	13419	40.9
Ethnicity																		
Malay	12963	54.8	13839	56.0	14272	55.2	16355	56.2	11693	58.6	14486	56.1	17039	56.9	18470	57.9	19150	58.4
Chinese	5418	22.9	5564	22.5	5761	22.3	6469	22.2	3848	19.3	5265	20.4	5812	19.4	6208	19.4	6223	19.0
Indian	2465	10.4	2485	10.1	2513	9.7	2881	9.9	1649	8.3	2271	8.8	2691	9.0	2686	8.4	2832	8.6
Others	2817	11.9	2808	11.4	2690	10.4	3338	11.5	2757	13.8	3781	14.7	4386	14.6	4434	13.9	4487	13.7
Unknown	0	0.0	17	0.1	628	2.4	38	0.1	3	0.0	0	0.0	37	0.1	124	0.4	112	0.3
Stroke subtypes																		
Ischemic	17280	73.0	17749	71.8	18313	70.8	20547	70.7	14748	73.9	18761	72.7	21383	71.4	23192	72.7	24109	73.5
Hemorrhagic	3812	16.1	4259	17.2	4803	18.6	5503	18.9	3182	15.9	4354	16.9	5573	18.6	5692	17.8	5653	17.2
Subarachnoid hemorrhage	438	1.9	450	1.8	525	2.0	605	2.1	399	2.0	627	2.4	764	2.5	698	2.2	693	2.1
Transient Ischemic Attack (TIA)	2133	9.0	2255	9.1	2223	8.6	2426	8.3	1621	8.1	2061	8.0	2245	7.5	2340	7.3	2349	7.2
Median length of hospital stay* (IQR)	3	(3)	3	(4)	3	(4)	3	(3)	3	(4)	3	(4)	3	(4)	3	(4)	3	(3)

* Length of stay includes the duration between hospital transfers.

More than half of the stroke patients were of Malay ethnicity. The proportion of patients with ischemic stroke, hemorrhagic stroke and subarachnoid hemorrhage remained almost constant over the years whereas those who were admitted for TIA were slightly reduced (9.0% in 2008 to 7.2% in 2016). On average, the median length of stay in the hospital for a stroke event was three days.

**Figure 2:**
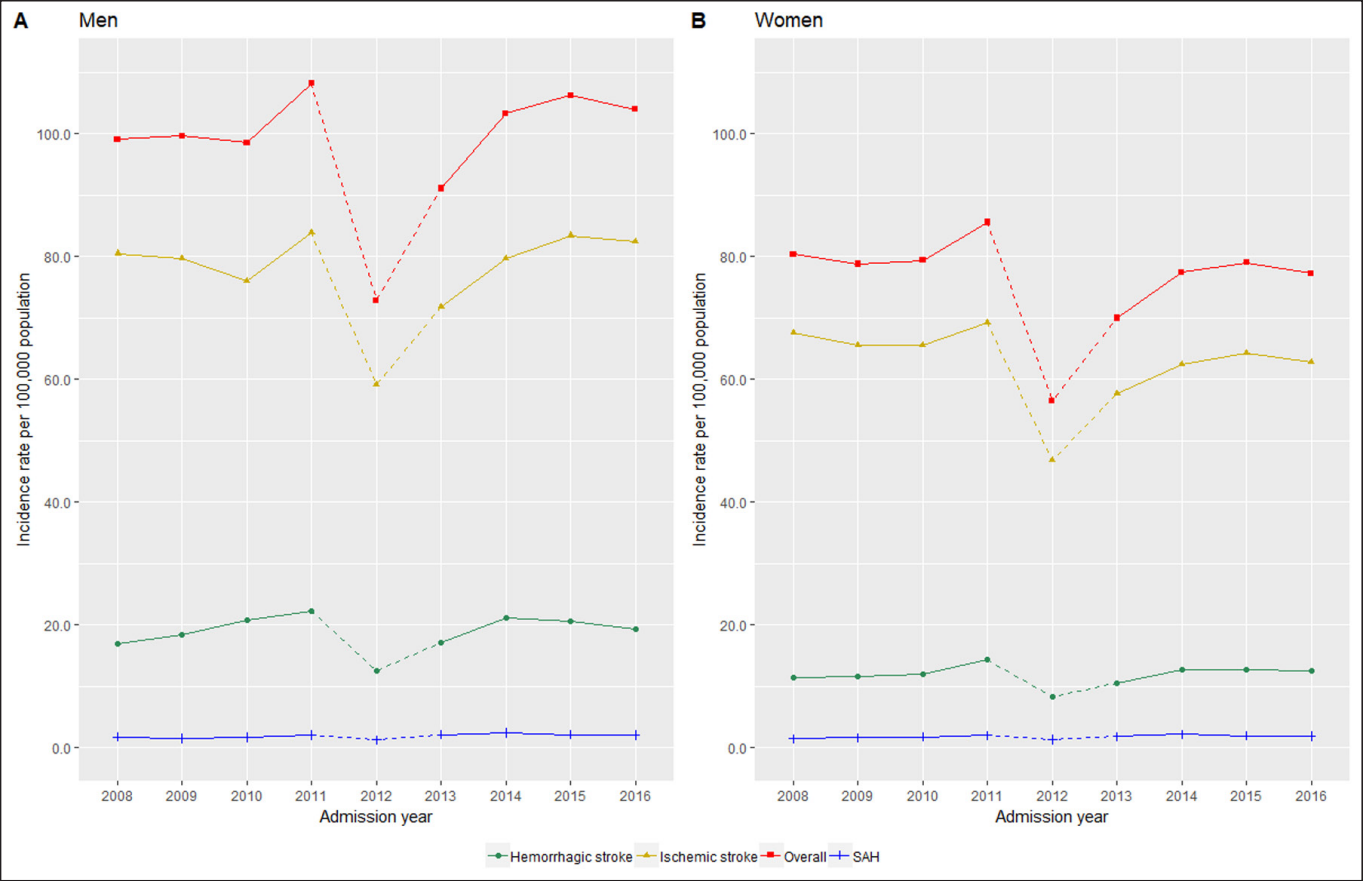
**Trends in the age-adjusted incidence for hospitalized stroke by its subtypes for A) men and B) women from year 2008 to 2016 in Malaysia (age and sex-adjusted to 2016 Malaysian population).** * Note: Dashed lines between 2012 and 2013 to indicate poor response rates as a result of starting a new web-based registration for hospital admissions in the country.

**Figure 3:**
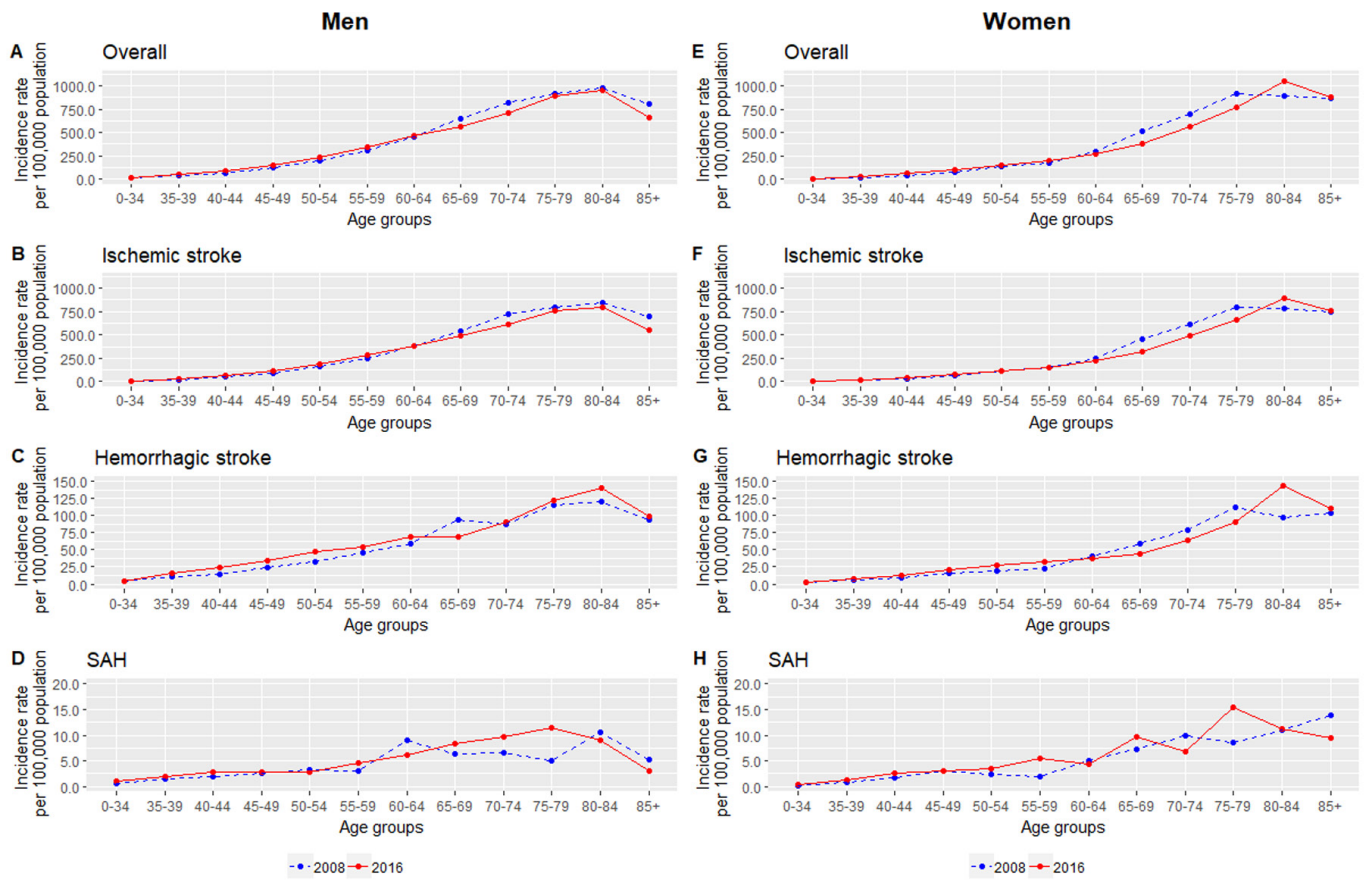
**Trends in the incidence of hospitalized stroke by age groups and subtypes for men (A–D) and women (E–H) for year 2008 and 2016 in Malaysia.** ICD-10 codes for overall stroke includes I60–I64 and G45, ischemic stroke (I63–I64 and G45), hemorrhagic stroke (I61–I62) and subarachnoid haemorrhage (I60).

Overall, the trend for age-standardized incidence of hospitalized stroke between 2008 and 2016 remained stable, despite an increase of 4.9% for men whereas in women, a fall of 3.8% (Figure 2). Age-standardized incidence were observed to be consistently higher in men, at 99.1 per 100,000 in 2008 and 103.9 per 100,000 population in 2016 compared to women (80.3 per 100,000 in 2008 and 77.2 per 100,000 in 2016). As displayed in Table S.1 and S.2, a substantial increase in stroke incidence among those aged 65 years and below was observed. Age-specific increases ranged from 2.7% to 53.3% among men whereas for women, the increase was between 3.9% to 50.4%. The rise was most significant in those between 35 to 49 years. Contrastingly, a drop was observed in people who are 65 years and older. This was similar in men and women although there was another increase for women after 80 years. This increment between 2008 and 2016 in the younger age groups was similarly highlighted in Figure 3.

**Figure 4:**
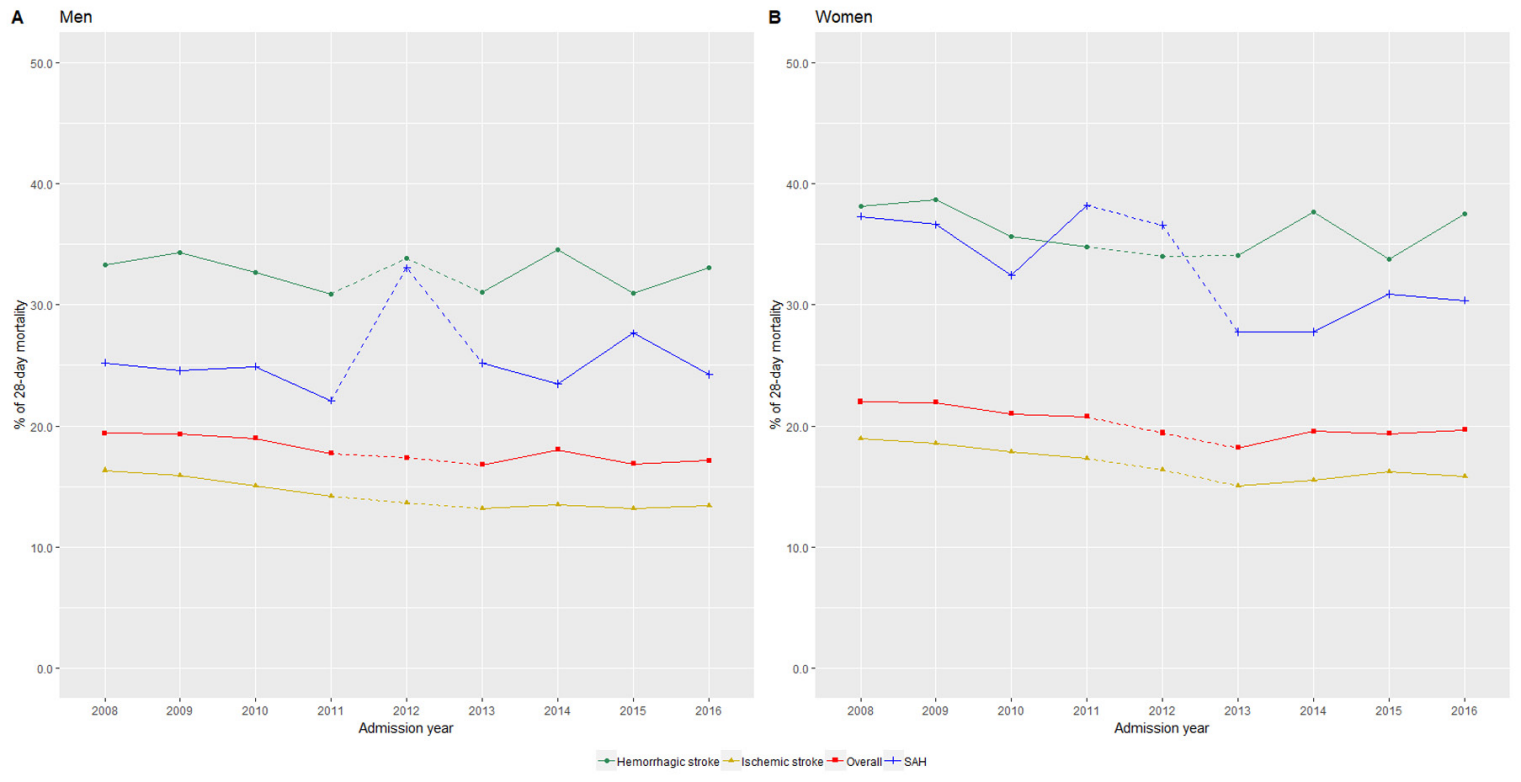
**Trends in the age-adjusted proportion of 28-day all-cause mortality from stroke by its subtypes for A) men and B) women from year 2008 to 2016 in Malaysia (age and sex-adjusted to 2016 Malaysian population).** * Note: Dashed lines between 2012 and 2013 to indicate a drop of response rates as a result of starting a new web-based registration for hospital admissions in the country.

In Figure 4, there was a significant drop in age-standardized proportions of 28-day all-cause mortality after an overall stroke for both men (-13.1%) and women (-10.6%). In contrast to the estimates for incidence, women were observed to have higher mortality (22.0% in 2008 and 19.7% in 2016) than men (19.4% in 2008 to 17.2% in 2016). Within specific age groups (Table S.3 and S.4), the change between 2008 and 2016 ranged from +9.4% to –25.2% with the largest change noted in men aged 45 to 49 years. For women, those between 60 to 64 years had the largest drop of 20.9%. Comparing 2008 and 2016, Figure 5 displays a substantial and consistent decrease in the proportions of 28-day all-cause mortality after a stroke.

**Figure 5:**
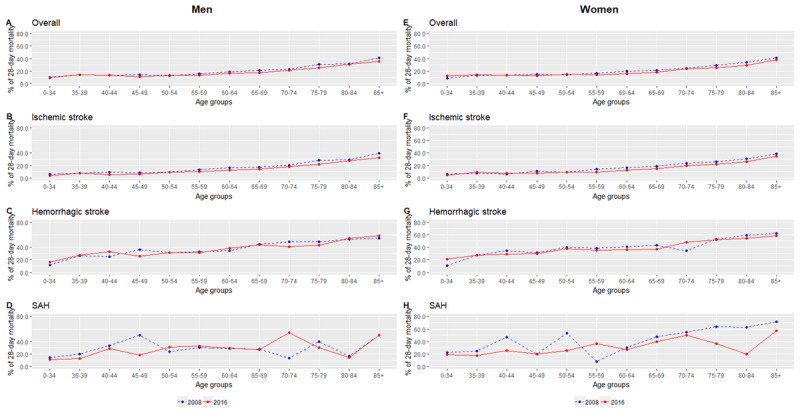
**Trends in the proportion of 28-day all-cause mortality from stroke by age groups and subtypes for men (A–D) and women (E–H) for year 2008 and 2016 in Malaysia.** ICD-10 codes for overall stroke includes I60–I64 and G45, ischemic stroke (I63–I64 and G45), hemorrhagic stroke (I61–I62) and subarachnoid haemorrhage (I60).

### Ischemic stroke

A rise in the trend was seen among younger age groups which include 1.9% to 2.6% for 35 to 39 years, 11.9% to 13.0% for 55 to 59 years, and 13.2% to 14.1% for 60 to 64 years (Table S.5). Proportions of yearly ischemic stroke admissions by sex, ethnic group and median length of stay were similar to overall stroke.

As displayed in Figure 2, an overall increase of 2.4% for men and a reduction of 6.9% for women was noted between 2008 and 2016. Tables S.6 and S.7 highlight an increase ranging from 1.4% to 61.3% for men below 65 years whereas for those above 65 years, there was a reduction ranging from –21.4 to –5.1%. This was consistently seen for women, with the exception that the reduction started much earlier from women of 50 years and above (Figure 3).

Similar to overall stroke, women consistently had a higher 28-day all-cause mortality after an ischemic stroke compared to men. There was a significant drop in the trend among men, with a fall of 18.1% from 2008 to 2016 and for women, lesser at 16.4% (Figure 4, Tables S.8 and S.9).

### Hemorrhagic stroke

Patients who were admitted for hemorrhagic stroke were younger than ischemic stroke patients with a mean age ranging from 53 to 55 years (Table S.10).

A rising trend for incidence of hemorrhagic stroke was noted in Figure 2, Tables S.11 and S.12, larger for men (16.8 per 100,000 in 2008 to 19.4 per 100,00 in 2016) compared to women (11.4 per 100,000 in 2008 to 12.5 per 100,000 in 2016). Despite little change in the proportions of 28-day all-cause mortality over the years, the proportions of patients who died after a hemorrhagic stroke were 2–2.5 times higher than ischemic stroke patients. Findings were similar for both sexes (Figure 4, Tables S.13 and S.14).

### Subarachnoid hemorrhage

Although the mean age for patients with subarachnoid hemorrhage was lower than other stroke subtypes (range: 49 to 51 years), there was no notable trend observed within specific age groups (Table S.15).

As shown in Figure 2, there was a slight increase in the incidence trend from 2008 to 2016. Changes between the years however, were fluctuating with age groups (Tables S.16 and S.17). Similarly, despite an overall drop in the trend of 28-day all-cause mortality after a subarachnoid haemorrhage, Figure 4 displays fluctuations across age groups for both men and women between 2008 and 2016. Tables S.18 and S.19 highlight a more prominent fall in mortality after subarachnoid hemorrhage at 18.6% among women compared to men (–3.8%).

## Discussion

The trend of hospitalized stroke incidence in Malaysia over the past decade remained stable, with a slight increase in men and a modest decline among women. Men consistently had higher incidence of stroke than women. Importantly, there was a substantial increase of stroke incidence among the younger population, especially among ischemic stroke patients. Despite a decline in the proportions of 28-day all-cause mortality after a stroke, women had a higher probability of dying within 28 days after a stroke at 1 in 5 compared to men at 1 in 6. The decline in mortality was largely contributed by its downward trend for ischemic stroke.

Owing to varying number of years assessed and methodologies used to estimate incidence between and within countries, diverging findings were observed from studies that provided temporal trends for stroke incidence. Direct comparisons of these findings to that of ours is therefore, difficult. Globally, an estimated decline of –8.1% in age-standardized stroke incidence was reported between 1990 and 2016 [17]. In the United States, stroke incidence dropped between 1998 and 2011 with an absolute decrease of 0.93 per 1000 population and similarly, a 33% drop in Sweden between 2001 and 2016 [18,19]. Instead, a stable stroke incidence for the Netherlands was seen from year 1997 to 2005 [20]. Within Asia, availability of temporal trends were largely limited to evidence from high-income countries. Singapore and Korea showed a decrease in their trends for stroke incidence; Singapore with a drop from 171.8 per 100,000 in 2007 to 155.2 in 2016 whereas in Korea, 105.8 per 100,000 in 2007 to 92.2 in 2013 [21,22]. The higher ratio of men with incident strokes compared to women is parallel to global findings [17], results across Asian countries as well as local estimates in selected areas [4,5,21] We expand the evidence; being among the first to show detailed age- and sex-specific trends from an Asian middle-income country, trends that are clearly different from high-income Asian countries.

One notable finding that should be highlighted is the increase in stroke incidence among the younger population below 65 years, which was substantially contributed by the increase in ischemic stroke. Worldwide, similar observations have increasingly been reported. The Global Burden of Disease Study showed a 25% increase of stroke incidence among young adults aged 20 to 64 years old from 1990 to 2010, which were mostly attributable to LMICs [1]. In Singapore, there was a similar upward trend in stroke incidence between those aged 30 to 60 years old from 2007 to 2016 [21]. Incidence of hospitalized stroke among Danish young adults was reported to increase with an annual change of 1.8% between 1994 and 2012 [23]. Likewise in the Netherlands, a significantly increasing trend of stroke incidence was observed among those aged between 35 to 64 years [20].

Multiple reasons have been postulated for the rise of incidence among young adults but evidence to support these reasons are yet to be established. The observed trend is possibly a reflection of a parallel increase in the burden of cardiovascular risk factors especially hypertension among young adults. The National Health and Morbidity Survey, a nationwide community survey in Malaysia which is conducted periodically, showed a slight increasing trend of the prevalence of hypertension among those aged 18–39 years (17.6% in 2006 to 18.9% in 2015) and 40–59 years (48.8% in 2006 to 49.5% in 2015) [24]. As for those 60 years and above, the prevalence of hypertension showed a downward trend from 73.1% in 2006 to 71.9% in 2015. Although awareness, treatment and control of the disease were reported to have increased in all age groups over the 10 years, it was evident that these improvements were modest in the those below 60 years of age in comparison to the older age group. The prevalence of awareness among hypertensives only increased from 39.1% in 2006 to 39.5% in 2015 among patients aged 40–59 years whereas in those 60 years and above, prevalence of awareness rose from 50.6% in 2006 to 57.2% in 2015. Likewise, the younger age group reported a rise in the control of treated hypertensives from 29.6% in 2006 to 36.7% in 2015 but a larger improvement was observed among those 60 years and above (24.3% in 2006 to 37.3% in 2015).

In addition to that, the proportion of Malaysians who have >40% 10-year cardiovascular risk was found to have increased among those aged 40–59 years old from 2006 to 2015 whereas a decline was observed for those above 60 years of age [25]. Despite a downward trend in the prevalence of Malaysians with undiagnosed cardiovascular risk factors between the year 2011 and 2015, the proportions remain significant for those aged between 35 to 64 years [26]. In 2015, the prevalence of undiagnosed dyslipidemia among these age groups ranged from 40.0 to 48.5%, hypertension (18.1–27.9%) and diabetes mellitus (9.5–12.4%).

Findings on the decreasing trend of 28-day all-cause mortality after a stroke is comparable to high-income countries. In Sweden, their 28-day all-cause mortality for stroke patients declined from 14.3% in 2001–2002 to 11.1% in 2015–2016 [19], whereas in the United States, 30-day all-cause mortality after an ischemic stroke decreased from 15.9% in 1988 to 12.7% in 2008 and hemorrhagic stroke, 44.7% to 39.3% [27]. Similarly in Singapore, with an Asian population, a decline in 30-day mortality specifically due to stroke was noted (10.4% in 2011 to 8.0% in 2016) [21].

Globally, a downward trend in mortality after a stroke, in particular those of ischemic origin is substantially attributable to improved management of stroke care. This ranges from control of cardiovascular risk factors, emphasis on the importance of secondary stroke prevention, better detection with improved diagnostic tools, introduction of reperfusion therapies to development of stroke systems of care such as acute stroke units, telemedicine and primary and comprehensive stroke centres [28]. The magnitude of such improvement however, is highly dependent on the availability of expertise and resources in respective countries. In Malaysia, the reduction in the 28-day all-cause mortality after a stroke is observed to be in line with the continuous development of stroke care in the country, in particular the hyperacute stroke care components which includes recommendations for use of acute reperfusion therapies and establishment of acute stroke units in some hospitals in this country [29,30]. This is supported with trend data from the Malaysian National Stroke Registry which reported that there were lesser stroke patients being discharged from the hospitals with poor clinical outcomes between 2009 compared to 2017 [6]. The proportion of stroke patients with functional dependence upon hospital discharge has dropped from 56.0% in 2009 to 45.8% in 2017. Proportion of overall deaths at discharge has also decreased from 20.7% in 2009 to 7.8% in 2017.

To the best of our knowledge, this is the first effort to contribute empirical local trends for stroke metrics in such large cohorts for Malaysia. Previous studies have either provided estimates for localized areas within Malaysia or made use of estimates from epidemiological models [4,5,7,8]. With data linkage methods, we were able to perform our analyses using population data sources. Despite not a nationwide estimation, this information provides a Malaysian baseline morbidity and mortality statistics for stroke, which is an essential tool to guide health policy planning. The admission records were available for almost all Ministry of Health hospitals which caters to nearly 70% of total hospital admissions in the country [31]. Future extrapolations to include stroke cases from private hospitals, university hospitals and non-hospitalized stroke patients is possible with the availability of this current information. We acknowledge our limitations which include an underestimation of estimates for year 2012 and 2013 due to poor response rates from participating hospitals as a result of a switch in data collection system from manual to web-based. Moreover, true estimates were underestimated due to restriction of data to Ministry of Health hospitals. For trends, assumptions were made that there were no temporal changes in referral patterns and mortality rates between Ministry of Health and other hospitals. Furthermore, possible confounders such as stroke severity, co-morbidities and socioeconomic statuses were not available and thus, their impact on outcome and trends could not be evaluated.

### Policy and research implications

Findings from this study carry important implications in strengthening local cardiovascular prevention and management of stroke care. Firstly, the non-declining trend of hospitalized stroke incidence for the past decade calls for an urgent need to identify better ways to improve the uptake and effectiveness of primary cardiovascular preventive strategies for the country. Optimal screening strategies should ideally be sex-specific with different age group targets, taking into account the increasing stroke incidence among young adults and higher stroke incidence in men compared to women. Barriers that potentially limit the effectiveness of current primary cardiovascular preventive programmes should be explored. In addition, use of cardiovascular risk assessment tools needs to be advocated. Recent evidence has shown that patients had a significant reduction in their modelled cardiovascular risks following provision of estimates from cardiovascular risk models to them [32].

Secondly, the downward trend for mortality after stroke may reflect the continuous efforts to improve the delivery of stroke care in Malaysia. Nevertheless, the considerably high mortality estimates in 2016 implies that more insistent actions should be pushed forward, in particular across Ministry of Health hospitals as these hospitals cover the largest bulk of hospital admissions in the country. Efforts to enhance stroke care which includes improving access to better diagnostic tools, providing up-to-date standardized hyperacute stroke care across the country, as well as establishing more acute stroke units and comprehensive stroke centres should be hastened. Importantly, research focusing on improving local stroke services and cost-effectiveness of these strategies is imperative.

In conclusion, the non-declining trend of stroke incidence is compounded with an increase among the younger population in Malaysia. Despite an overall decline in the proportions of mortality after stroke, there was still on average one death for every five strokes in women and in men, one in every six. Effective strategies to strengthen cardiovascular prevention and management of stroke care in the country are thus, urgently warranted.

## Data Accessibility Statement

Data that support the findings of this study are available from Health Informatics Centre, Ministry of Health Malaysia and the National Registration Department of Malaysia. Due to ethical and legal restrictions, the data is not available publicly. Requests for the use of the data should be made to the corresponding author with permission from data owners.

## Additional File

The additional file for this article can be found as follows:

10.5334/gh.791.s1 Supplementary Results. Tables S1–S19.
