# Supplementary material for: Trends of Stroke Incidence and 28-Day All-Cause Mortality after a Stroke in Malaysia: A Linkage of National Data Sources
Source: Glob Heart. 2021 May 26;16(1):39. doi: 10.5334/gh.791 (PMC8162294; doi:10.5334/gh.791)
Supplement: Supplementary Results. — Tables S1–S19. [file gh-16-1-791-s1.pdf]

Supplementary Results

Table S1. Incidence of hospitalized stroke for men between year 2008 and 2016 in Malaysia*

Age groups	2008	2009	2010	2011	2012	2013	2014	2015	2016	†Change from 2008 - 2016	‡Trends (p-value)
0-34	8.8	9.7	9.4	10.4	6.5	8.3	10.5	10.1	9.7	10.4	0.47
35-39	33.6	38.0	40.1	42.7	29.3	39.2	50.4	51.0	51.5	53.3	0.02
40-44	63.9	69.2	70.9	84.7	51.1	70.0	80.1	91.4	91.3	43.0	0.05
45-49	119.3	123.3	120.1	129.3	91.3	123.7	143.9	148.5	152.6	27.9	0.02
50-54	201.2	206.2	204.6	231.3	152.7	186.8	219.7	230.5	237.7	18.1	0.18
55-59	303.3	315.0	319.6	340.9	234.2	289.0	328.8	334.3	345.3	13.8	0.12
60-64	447.8	420.1	429.7	470.1	314.0	397.4	437.0	452.8	460.1	2.7	0.47
65-69	642.3	628.1	606.5	616.9	435.7	526.3	589.3	601.8	565.2	-12.0	0.08
70-74	822.2	811.5	776.3	863.0	570.5	735.3	790.3	817.0	710.0	-13.6	0.35
75-79	915.3	950.7	877.3	1039.3	699.8	821.4	925.2	942.6	888.7	-2.9	0.92
80-84	975.4	863.5	918.2	1041.1	736.7	833.6	971.6	953.2	947.2	-2.9	0.92
85+	802.1	790.7	794.0	872.6	591.6	744.4	692.6	743.3	655.4	-18.3	0.08
Total	85.2	87.5	88.5	99.1	68.1	87.1	101.1	106.1	103.9	22.0	0.05
Total (age-adjusted to 2016 Malaysian population)	99.1	99.6	98.5	108.1	72.8	91.0	103.3	106.2	103.9	4.9	0.47
Total (age adjusted to WHO standard world population)	125.1	125.1	123.5	135.9	91.8	114.2	128.8	132.4	128.7	2.9	0.60

*stroke includes ICD-10 codes from I60 – I64 and G45
†change is defined as = (2016 – 2008/2008) x 100%
‡Mann-Kendall trend test

Table S2. Incidence of hospitalized stroke for women between year 2008 and 2016 in Malaysia*

Age groups	2008	2009	2010	2011	2012	2013	2014	2015	2016	†Change from 2008 - 2016	‡Trends (p-value)
0-34	4.3	5.0	4.4	4.9	3.0	3.9	4.9	4.8	5.2	18.6	0.60
35-39	19.7	21.3	20.3	22.2	15.7	20.1	28.3	29.0	29.6	50.4	0.05
40-44	41.7	40.4	43.3	48.9	33.3	42.5	47.7	52.4	57.6	38.1	0.05
45-49	78.1	78.5	81.9	92.2	63.1	83.0	88.4	90.9	96.9	24.1	0.03
50-54	138.3	133.9	140.3	148.0	106.0	128.5	143.5	141.8	143.7	3.9	0.35
55-59	178.4	183.4	203.4	208.1	137.5	160.1	196.5	197.5	193.1	8.2	0.75
60-64	291.2	273.9	284.0	301.8	198.4	240.3	282.8	285.9	269.9	-7.3	0.60
65-69	518.4	468.7	449.6	471.0	311.5	352.5	389.6	400.7	379.6	-26.8	0.12
70-74	699.6	667.0	666.8	671.5	449.7	600.8	637.8	602.6	562.6	-19.6	0.05
75-79	911.6	888.1	899.5	997.7	673.8	789.7	804.2	862.2	767.8	-15.8	0.18
80-84	885.4	886.5	926.9	1082.5	682.3	946.5	1009.7	1052.6	1050.4	18.6	0.08
85+	869.3	1002.0	850.6	1059.0	577.2	772.2	734.9	826.9	881.5	1.4	0.60
Total	67.6	67.7	69.9	76.9	52.0	66.0	74.7	78.1	77.2	14.3	0.12
Total (age-adjusted to 2016 Malaysian population)	80.3	78.8	79.3	85.5	56.5	70.0	77.4	79.0	77.2	-3.8	0.35
Total (age adjusted to WHO standard world population)	95.9	94.0	94.6	102.2	67.4	83.8	92.1	94.0	91.7	-4.4	0.35

*stroke includes ICD-10 codes from I60 – I64 and G45
†change is defined as = (2016 – 2008/2008) x 100%
‡Mann-Kendall trend test

Table S3. Proportion of 28-day all-cause mortality from stroke for men between year 2008 and 2016 in Malaysia*

Age groups	2008	2009	2010	2011	2012	2013	2014	2015	2016	†Change from 2008 - 2016	‡Trends (p-value)
0-34	9.5	10.6	11.1	10.1	10.5	9.9	11.9	10.9	10.4	9.4	0.60
35-39	14.2	12.4	13.3	13.5	11.2	14.0	14.3	13.0	14.2	-0.1	0.60
40-44	13.4	13.7	13.6	11.2	12.2	14.3	12.3	13.4	13.2	-1.7	0.75
45-49	14.5	16.1	15.7	14.7	13.0	13.2	12.2	13.1	10.9	-25.2	0.02
50-54	13.1	15.3	16.2	14.1	14.2	10.9	12.8	13.6	13.6	4.1	0.47
55-59	16.3	16.7	15.7	15.1	13.6	14.2	14.7	13.9	13.6	-16.5	0.02
60-64	19.2	18.2	17.7	17.4	16.6	14.5	16.4	15.3	16.5	-14.5	0.01
65-69	21.5	21.2	20.1	18.3	18.2	17.7	20.2	17.1	17.7	-17.3	0.02
70-74	23.3	21.8	22.7	22.2	21.6	21.2	22.5	21.0	21.2	-8.9	0.03
75-79	30.7	28.8	26.6	24.0	25.6	24.8	26.4	22.9	25.3	-17.8	0.05
80-84	32.1	33.4	30.8	29.6	30.0	32.9	32.5	29.3	31.3	-2.3	0.47
85+	41.0	37.4	40.4	37.5	38.1	33.6	40.8	38.8	36.0	-12.1	0.47
Total	19.6	19.4	19.1	17.9	17.6	17.0	18.1	16.9	17.2	-12.3	0.01
Total (age-adjusted to 2016 stroke patients)	19.4	19.3	19.0	17.7	17.4	16.8	18.1	16.9	17.2	-13.1	**0.02**

*stroke includes ICD-10 codes from I60 – I64 and G45

†change is defined as = (2016 – 2008/2008) x 100%

‡Mann-Kendall trend test

Table S4. Proportion of 28-day all-cause mortality from stroke for women between year 2008 and 2016 in Malaysia*

Age groups	2008	2009	2010	2011	2012	2013	2014	2015	2016	Change from 2008 - 2016	Trends (p-value)
0-34	8.9	12.3	10.0	11.9	10.3	10.1	10.0	11.2	12.6	42.6	0.35
35-39	13.9	16.1	16.3	13.3	16.5	11.7	12.0	12.7	14.5	4.0	0.60
40-44	13.7	12.8	12.4	14.1	8.5	8.2	9.9	12.7	13.6	-0.8	0.60
45-49	15.3	15.1	14.5	14.5	15.2	11.7	12.4	11.9	12.6	-17.1	0.08
50-54	14.7	16.3	15.4	13.0	15.2	12.3	13.0	14.7	14.8	0.5	0.47
55-59	17.1	18.2	14.3	16.2	16.7	13.2	13.0	14.1	14.7	-13.7	0.12
60-64	20.0	18.9	18.7	19.1	18.3	16.5	17.1	16.5	15.8	-20.9	0.005
65-69	21.7	21.0	19.4	19.1	17.9	17.8	20.0	18.4	18.1	-16.5	0.05
70-74	25.1	23.2	24.0	23.6	20.8	20.6	23.2	22.2	23.7	-5.8	0.35
75-79	29.3	30.1	28.3	28.8	25.6	25.9	26.4	25.9	25.7	-12.4	0.05
80-84	34.0	34.0	34.2	32.4	27.9	28.2	31.7	32.1	29.7	-12.6	0.12
85+	41.8	39.3	40.6	37.3	34.3	35.5	38.6	34.7	38.1	-8.8	0.12
Total	22.2	22.2	21.1	21.1	19.5	18.6	19.6	19.5	19.7	-11.6	0.05
Total (age-adjusted to 2016 stroke patients)	22.0	21.9	21.0	20.8	19.4	18.2	19.6	19.4	19.7	-10.6	**0.03**

*stroke includes ICD-10 codes from I60 – I64 and G45

†change is defined as = (2016 – 2008/2008) x 100%

‡Mann-Kendall trend test

Table S5. Baseline characteristics of hospital admissions for ischemic stroke from 2008 to 2016*

	Year																	
	2008 (n)		2009 (n)		2010 (n)		2011 (n)		2012 (n)		2013 (n)		2014 (n)		2015 (n)		2016 (n)	
Hospital admissions for stroke (excluding transfers)	19413		20004		20536		22973		16369		20822		23628		25532		26458	
Baseline characteristics	n	(%)	n	(%)	n	(%)	n	(%)	n	(%)	n	(%)	n	(%)	n	(%)	n	(%)
Mean age (SD)	63	(14)	63	(14)	63	(14)	63	(14)	63	(14)	63	(14)	62	(14)	63	(14)	62	(14)
Age groups																		
0-34	550	2.8	615	3.1	539	2.6	566	2.5	454	2.8	544	2.6	673	2.8	697	2.7	768	2.9
35-39	366	1.9	408	2.0	437	2.1	463	2.0	356	2.2	413	2.0	575	2.4	606	2.4	687	2.6
40-44	769	4.0	803	4.0	794	3.9	964	4.2	642	3.9	819	3.9	953	4.0	1122	4.4	1197	4.5
45-49	1320	6.8	1376	6.9	1394	6.8	1556	6.8	1160	7.1	1494	7.2	1661	7.0	1749	6.9	1910	7.2
50-54	2031	10.5	2054	10.3	2171	10.6	2485	10.8	1787	10.9	2175	10.4	2538	10.7	2662	10.4	2797	10.6
55-59	2314	11.9	2441	12.2	2656	12.9	2853	12.4	2019	12.3	2607	12.5	3042	12.9	3285	12.9	3444	13.0
60-64	2567	13.2	2635	13.2	2762	13.4	3173	13.8	2222	13.6	2846	13.7	3287	13.9	3519	13.8	3718	14.1
65-69	2869	14.8	2813	14.1	2761	13.4	2998	13.1	2244	13.7	2813	13.5	3266	13.8	3577	14.0	3655	13.8
70-74	2798	14.4	2906	14.5	2881	14.0	3156	13.7	2093	12.8	2744	13.2	2908	12.3	3077	12.1	3060	11.6
75-79	1950	10.0	1986	9.9	2048	10.0	2385	10.4	1803	11.0	2256	10.8	2531	10.7	2756	10.8	2634	10.0
80-84	1177	6.1	1187	5.9	1309	6.4	1461	6.4	1001	6.1	1244	6.0	1354	5.7	1485	5.8	1584	6.0
85+	702	3.6	780	3.9	784	3.8	913	4.0	588	3.6	867	4.2	840	3.6	997	3.9	1004	3.8
Sex																		
Men	10942	56.4	11411	57.0	11458	55.8	13105	57.0	9433	57.6	11915	57.2	13714	58.0	14889	58.3	15507	58.6
Women	8471	43.6	8593	43.0	9078	44.2	9868	43.0	6936	42.4	8907	42.8	9914	42.0	10643	41.7	10951	41.4

Ethnicity																		
Malay	10805	55.7	11408	57.0	11644	56.7	13338	58.1	9879	60.4	11970	57.5	13830	58.5	15141	59.3	15704	59.4
Chinese	4286	22.1	4370	21.8	4384	21.3	4810	20.9	3045	18.6	4061	19.5	4302	18.2	4746	18.6	4825	18.2
Indian	2186	11.3	2178	10.9	2148	10.5	2467	10.7	1461	8.9	1976	9.5	2318	9.8	2360	9.2	2526	9.5
Others	2136	11.0	2036	10.2	1954	9.5	2335	10.2	1981	12.1	2815	13.5	3152	13.3	3193	12.5	3309	12.5
Unknown	0	0.0	12	0.1	406	2.0	23	0.1	3	0.0	0	0.0	26	0.1	92	0.4	94	0.4
Median length of hospital stay† (IQR)	3	(3)	3	(3)	3	(3)	3	(3)	3	(4)	3	(4)	2	(4)	3	(4)	3	(3)

*ischemic stroke includes ICD-10 codes from I63 – I64 and G45 †length of stay includes the duration between hospital transfers

Table S6. Incidence of hospitalized ischemic stroke for men between year 2008 and 2016 in Malaysia*

Age groups	2008	2009	2010	2011	2012	2013	2014	2015	2016	†Change from 2008 - 2016	‡Trends (p-value)
0-34	3.4	3.6	3.1	3.4	2.8	3.2	4.0	4.0	4.1	22.9	0.18
35-39	20.9	24.6	24.9	26.9	20.1	24.1	30.2	31.1	33.7	61.3	0.03
40-44	48.3	50.6	47.4	60.4	36.8	47.6	55.1	65.3	64.1	32.6	0.18
45-49	93.3	93.2	88.0	97.3	73.5	92.8	102.5	110.2	115.7	24.1	0.12
50-54	166.2	168.0	161.3	186.9	124.1	148.1	170.5	183.9	187.9	13.1	0.25
55-59	253.8	265.2	256.9	270.6	191.0	236.9	266.7	270.9	286.6	12.9	0.12
60-64	379.2	351.2	348.1	384.8	263.1	333.0	364.7	372.3	384.6	1.4	0.75
65-69	542.6	534.8	497.8	515.5	382.8	442.2	488.4	514.0	487.9	-10.1	0.12
70-74	727.8	703.1	653.7	719.6	487.7	621.2	664.6	703.8	609.3	-16.3	0.25
75-79	794.8	812.4	730.0	860.4	612.3	703.4	786.7	800.4	754.3	-5.1	0.75
80-84	843.9	738.5	789.3	876.8	639.5	693.6	785.1	788.2	797.9	-5.4	0.92
85+	703.2	666.7	671.3	709.9	507.7	664.6	578.7	634.6	552.8	-21.4	0.08
Total	68.5	69.3	67.7	76.3	55.0	68.4	77.8	83.3	82.4	20.4	0.12
Total (age-adjusted to 2016 Malaysian population)	80.5	79.7	76.0	83.9	59.1	71.8	79.7	83.4	82.4	2.4	0.92
Total (age adjusted to WHO standard world population)	103.1	101.5	96.9	107.0	75.5	91.4	101.0	105.6	103.5	0.4	0.92

*ischemic stroke includes ICD-10 codes from I63 – I64 and G45

†change is defined as = (2016 – 2008/2008) x 100%

‡Mann-Kendall trend test

Table S7. Incidence of hospitalized ischemic stroke for women between year 2008 and 2016 in Malaysia*

Age groups	2008	2009	2010	2011	2012	2013	2014	2015	2016	†Change from 2008 - 2016	‡Trends (p-value)
0-34	2.2	2.5	2.1	2.1	1.6	1.9	2.4	2.5	2.7	21.3	0.60
35-39	13.7	14.4	14.0	14.9	10.4	13.1	19.5	20.1	20.4	48.7	0.08
40-44	31.4	30.5	31.5	34.6	25.0	31.7	35.7	39.4	41.7	32.9	0.02
45-49	59.6	62.2	63.7	68.9	47.3	62.6	66.2	67.3	72.3	21.2	0.05
50-54	116.5	105.4	111.7	116.5	87.2	103.7	114.4	108.8	113.6	-2.5	0.92
55-59	154.8	152.4	173.2	170.0	112.0	133.8	158.3	161.4	154.6	-0.2	0.92
60-64	244.7	239.3	236.0	245.1	167.8	201.1	234.3	241.7	228.2	-6.8	0.35
65-69	453.0	406.4	381.9	398.0	268.0	305.4	326.0	340.9	325.2	-28.2	0.08
70-74	611.3	576.2	567.1	578.2	386.4	520.1	542.4	521.9	491.1	-19.7	0.05
75-79	790.8	778.8	788.5	865.1	593.0	683.6	677.4	739.6	661.7	-16.3	0.12
80-84	777.6	760.0	809.2	910.1	606.9	831.9	858.7	892.4	895.7	15.2	0.08
85+	752.5	872.5	748.7	897.6	498.4	659.8	623.6	706.7	761.6	1.2	0.60
Total	56.4	55.9	57.4	61.9	43.0	54.3	60.2	63.5	62.8	11.4	0.18
Total (age-adjusted to 2016 Malaysian population)	67.5	65.5	65.5	69.2	46.9	57.7	62.4	64.3	62.8	-6.9	0.35
Total (age adjusted to WHO standard world population)	81.0	78.7	78.6	83.4	56.3	69.6	74.8	77.1	75.2	-7.2	0.25

*ischemic stroke includes ICD-10 codes from I63 – I64 and G45

†change is defined as = (2016 – 2008/2008) x 100%

‡Mann-Kendall trend test

Table S8. Proportion of 28-day all-cause mortality from ischemic stroke for men between year 2008 and 2016 in Malaysia*

Age groups	2008	2009	2010	2011	2012	2013	2014	2015	2016	†Change from 2008 - 2016	‡Trends (p-value)
0-34	6.1	5.6	8.5	7.6	4.1	5.1	4.2	5.5	4.1	-32.0	0.12
35-39	7.5	4.9	6.1	5.8	5.3	6.3	5.0	5.4	8.1	7.8	0.92
40-44	9.2	7.6	8.1	5.2	6.2	7.0	5.8	7.0	5.4	-41.9	0.08
45-49	8.4	10.3	7.9	9.0	7.5	7.9	7.0	8.1	6.4	-23.3	0.08
50-54	9.1	11.0	11.1	9.6	9.2	6.2	7.9	9.6	9.1	0.8	0.47
55-59	13.0	12.5	12.1	11.2	10.0	11.1	9.7	10.1	10.1	-22.6	0.01
60-64	16.7	14.4	13.5	14.1	12.6	11.5	12.2	12.2	12.5	-25.3	0.03
65-69	17.4	18.3	16.2	14.7	15.0	14.4	15.0	14.1	14.1	-19.1	0.02
70-74	20.4	19.4	19.6	19.9	18.5	17.8	18.8	17.2	17.9	-12.4	0.03
75-79	28.1	25.8	23.1	20.7	22.2	21.2	22.6	19.4	22.2	-21.0	0.08
80-84	29.5	31.0	28.7	26.2	28.0	29.2	27.9	24.9	27.6	-6.5	0.05
85+	39.2	35.3	36.1	33.7	33.5	30.9	35.9	34.6	32.2	-18.0	0.08
Total	16.7	16.1	15.4	14.5	14.0	13.5	13.7	13.3	13.4	-19.6	0.00
Total (age-adjusted to 2016 ischemic stroke patients)	16.3	15.9	15.1	14.2	13.7	13.2	13.5	13.2	13.4	-18.1	0.002

*ischemic stroke includes ICD-10 codes from I63 – I64 and G45

†change is defined as = (2016 – 2008/2008) x 100%

‡Mann-Kendall trend test

Table S9. Proportion of 28-day all-cause mortality from ischemic stroke for women between year 2008 and 2016 in Malaysia*

Age groups	2008	2009	2010	2011	2012	2013	2014	2015	2016	†Change from 2008 - 2016	‡Trends (p-value)
0-34	5.9	7.1	4.3	6.6	3.8	4.1	4.5	6.5	4.9	-16.1	0.75
35-39	7.9	9.0	7.7	8.6	9.7	3.5	4.2	4.6	9.1	15.4	0.92
40-44	6.5	8.6	7.7	8.6	5.9	2.5	6.1	7.9	8.2	26.2	0.92
45-49	10.7	9.7	9.1	9.6	10.3	7.4	6.9	7.2	7.5	-29.8	0.05
50-54	9.8	10.1	11.3	9.1	12.2	7.7	8.6	10.0	9.3	-5.3	0.60
55-59	14.1	14.3	11.1	12.0	13.1	10.1	7.7	10.1	9.6	-32.0	0.03
60-64	16.4	15.8	14.4	14.8	14.2	12.9	13.1	13.2	12.4	-24.9	0.005
65-69	18.6	17.9	16.0	16.1	14.7	15.0	15.9	15.8	15.0	-19.3	0.05
70-74	23.4	20.5	20.5	19.9	18.3	17.8	18.5	19.1	20.1	-14.4	0.18
75-79	25.8	26.8	26.3	25.3	22.3	21.9	22.0	23.1	21.9	-15.2	0.03
80-84	30.6	30.4	31.6	28.8	25.3	26.1	27.6	29.0	26.3	-14.2	0.18
85+	38.7	36.9	39.1	34.8	32.4	33.5	35.4	32.1	35.0	-9.5	0.12
Total	19.2	18.9	18.0	17.7	16.5	15.4	15.5	16.4	15.9	-17.5	0.01
Total (age-adjusted to 2016 ischemic stroke patients)	19.0	18.6	17.9	17.3	16.4	15.1	15.5	16.2	15.9	-16.4	0.01

*ischemic stroke includes ICD-10 codes from I63 – I64 and G45

†change is defined as = (2016 – 2008/2008) x 100%

‡Mann-Kendall trend test

Table S10. Baseline characteristics of hospital admissions for hemorrhagic stroke from 2008 to 2016

	Year																	
	2008 (n)		2009 (n)		2010 (n)		2011 (n)		2012 (n)		2013 (n)		2014 (n)		2015 (n)		2016 (n)	
Hospital admissions for stroke (excluding transfers)	3812		4259		4803		5503		3182		4354		5573		5692		5653	
Baseline characteristics	n	%	n	%	n	%	n	%	n	%	n	%	n	%	n	%	n	%
Mean age (SD)	54	(20)	53	(20)	54	(19)	54	(20)	55	(18)	55	(18)	55	(18)	55	(18)	55	(18)
Age groups																		
0-34	646	16.9	781	18.3	741	15.4	859	15.6	423	13.3	573	13.2	762	13.7	730	12.8	731	12.9
35-39	172	4.5	199	4.7	216	4.5	214	3.9	143	4.5	217	5.0	283	5.1	314	5.5	302	5.3
40-44	214	5.6	242	5.7	308	6.4	338	6.1	196	6.2	311	7.1	349	6.3	381	6.7	406	7.2
45-49	332	8.7	363	8.5	412	8.6	464	8.4	274	8.6	417	9.6	541	9.7	567	10.0	533	9.4
50-54	386	10.1	446	10.5	506	10.5	575	10.4	352	11.1	460	10.6	620	11.1	643	11.3	665	11.8
55-59	387	10.2	442	10.4	517	10.8	622	11.3	409	12.9	489	11.2	640	11.5	658	11.6	665	11.8
60-64	403	10.6	403	9.5	548	11.4	626	11.4	355	11.2	481	11.0	603	10.8	622	10.9	635	11.2
65-69	427	11.2	435	10.2	505	10.5	508	9.2	300	9.4	416	9.6	541	9.7	568	10.0	497	8.8
70-74	334	8.8	388	9.1	446	9.3	507	9.2	312	9.8	393	9.0	457	8.2	410	7.2	420	7.4
75-79	271	7.1	271	6.4	311	6.5	393	7.1	223	7.0	316	7.3	393	7.1	402	7.1	394	7.0
80-84	150	3.9	181	4.2	177	3.7	242	4.4	112	3.5	175	4.0	245	4.4	245	4.3	248	4.4
85+	90	2.4	108	2.5	116	2.4	155	2.8	83	2.6	106	2.4	139	2.5	152	2.7	157	2.8
Sex																		
Men	2378	62.4	2702	63.4	3112	64.8	3473	63.1	1978	62.2	2782	63.9	3584	64.3	3614	63.5	3511	62.1
Women	1434	37.6	1557	36.6	1691	35.2	2030	36.9	1204	37.8	1572	36.1	1989	35.7	2078	36.5	2142	37.9

Ethnicity																		
Malay	1926	50.5	2210	51.9	2357	49.1	2679	48.7	1630	51.2	2200	50.5	2844	51.0	2971	52.2	3083	54.5
Chinese	1019	26.7	1070	25.1	1256	26.2	1518	27.6	696	21.9	1034	23.7	1331	23.9	1304	22.9	1244	22.0
Indian	253	6.6	280	6.6	331	6.9	386	7.0	165	5.2	256	5.9	335	6.0	296	5.2	256	4.5
Others	614	16.1	696	16.3	671	14.0	907	16.5	691	21.7	864	19.8	1054	18.9	1096	19.3	1055	18.7
Unknown	0	0.0	3	0.1	188	3.9	13	0.2	0	0.0	0	0.0	9	0.2	25	0.4	15	0.3
Median length of hospital stay† (IQR)	3	(6)	3	(6)	3	(6)	3	(5)	3	(6)	3	(6)	3	(6)	4	(6)	4	(6)

*hemorrhagic stroke includes ICD-10 codes from I61 – I62 †length of stay includes the duration between hospital transfers

Table S11. Incidence of hospitalized hemorrhagic stroke for men between year 2008 and 2016 in Malaysia*

Age groups	2008	2009	2010	2011	2012	2013	2014	2015	2016	†Change from 2008 - 2016	‡Trends (p-value)
0-34	4.7	5.5	5.5	6.2	3.0	4.1	5.5	5.0	4.6	-2.2	0.75
35-39	11.1	11.9	14.2	14.0	8.5	13.7	17.2	17.6	15.7	41.5	0.08
40-44	13.5	16.4	21.5	21.4	12.6	20.6	22.6	23.6	24.4	80.5	0.03
45-49	23.5	27.5	29.5	29.2	15.5	27.3	37.0	36.5	34.0	44.6	0.18
50-54	31.8	35.5	40.6	42.0	26.9	34.3	45.1	42.7	46.9	47.5	0.05
55-59	46.5	47.7	59.0	64.5	39.3	47.6	55.5	59.4	54.2	16.5	0.47
60-64	59.7	64.3	76.1	79.5	48.1	58.4	68.7	73.2	69.4	16.3	0.60
65-69	93.4	89.5	104.3	92.6	47.9	77.6	89.0	83.4	69.1	-26.1	0.08
70-74	87.8	99.5	111.1	133.6	78.0	102.5	114.7	102.6	91.1	3.7	0.75
75-79	115.5	125.7	140.9	171.0	84.4	107.9	127.6	126.8	122.8	6.3	0.92
80-84	121.1	115.1	121.1	151.7	86.2	126.0	174.6	151.3	140.3	15.9	0.18
85+	93.6	113.7	120.4	155.7	83.9	77.7	106.3	103.4	99.5	6.4	0.60
Total	15.1	16.8	19.2	20.8	11.9	16.6	20.9	20.7	19.4	28.5	0.25
Total (age-adjusted to 2016 Malaysian population)	16.8	18.4	20.8	22.2	12.5	17.1	21.1	20.6	19.4	15.0	0.60
Total (age adjusted to WHO standard world population)	20.0	21.8	24.6	26.6	14.9	20.4	25.0	24.3	22.8	14.1	0.60

*hemorrhagic stroke includes ICD-10 codes from I61 – I62

†change is defined as = (2016 – 2008/2008) x 100%

‡Mann-Kendall trend test

Table S12. Incidence of hospitalized hemorrhagic stroke for women between year 2008 and 2016 in Malaysia*

Age groups	2008	2009	2010	2011	2012	2013	2014	2015	2016	†Change from 2008 - 2016	‡Trends (p-value)
0-34	1.9	2.2	1.9	2.3	1.1	1.6	1.8	1.8	2.0	8.9	0.75
35-39	5.2	6.4	5.4	5.9	4.0	5.8	6.9	7.8	7.9	51.2	0.05
40-44	8.7	7.7	9.4	12.2	6.6	9.5	10.3	11.0	13.3	53.2	0.05
45-49	15.4	13.3	15.9	20.5	14.1	17.6	19.6	20.6	21.6	40.2	0.02
50-54	19.4	25.8	25.0	28.4	16.7	21.4	24.7	29.2	26.6	37.5	0.35
55-59	21.7	25.5	26.2	34.1	22.5	21.9	32.9	31.4	33.0	52.1	0.18
60-64	41.4	30.6	42.8	49.0	26.2	34.6	42.2	37.5	37.3	-9.9	0.92
65-69	58.3	56.1	59.8	65.3	37.2	39.2	53.0	52.4	44.8	-23.1	0.25
70-74	78.4	79.9	84.9	79.8	56.8	68.2	82.9	70.4	64.8	-17.4	0.35
75-79	112.2	95.9	102.1	119.4	74.4	92.9	111.2	111.8	90.6	-19.3	0.60
80-84	96.7	122.6	104.1	162.6	61.8	102.1	138.9	144.2	143.5	48.4	0.25
85+	103.0	113.7	96.7	142.4	69.1	100.6	105.8	113.7	110.4	7.2	0.75
Total	9.9	10.3	10.9	13.2	7.8	10.0	12.4	12.7	12.5	26.7	0.18
Total (age-adjusted to 2016 Malaysian population)	11.4	11.6	12.1	14.3	8.3	10.4	12.8	12.8	12.5	9.9	0.47
Total (age adjusted to WHO standard world population)	13.2	13.5	13.9	16.6	9.6	12.1	14.8	14.8	14.4	8.8	0.47

*hemorrhagic stroke includes ICD-10 codes from I61 – I62

†change is defined as = (2016 – 2008/2008) x 100%

‡Mann-Kendall trend test

Table S13. Proportion of 28-day all-cause mortality from hemorrhagic stroke for men between year 2008 and 2016 in Malaysia*

Age groups	2008	2009	2010	2011	2012	2013	2014	2015	2016	†Change from 2008 - 2016	‡Trends (p-value)
0-34	11.3	13.6	12.4	12.0	16.1	14.2	18.8	14.9	16.1	42.9	0.03
35-39	26.3	26.9	26.8	27.6	24.2	26.8	31.1	25.7	27.4	4.3	0.67
40-44	25.4	29.2	25.8	26.5	28.1	28.1	27.2	27.5	33.2	30.9	0.18
45-49	35.9	36.9	37.5	32.7	36.6	30.0	26.0	27.5	26.1	-27.4	0.03
50-54	31.6	35.0	35.8	33.9	38.0	29.6	28.9	28.2	31.1	-1.7	0.18
55-59	33.2	39.7	30.2	30.9	29.0	28.8	35.7	31.6	31.3	-5.9	0.60
60-64	34.4	39.4	36.2	33.1	37.9	30.8	38.1	30.0	38.1	10.7	0.92
65-69	44.7	38.0	39.7	37.1	39.7	36.5	48.4	35.0	43.7	-2.4	0.60
70-74	49.1	39.0	40.4	34.6	40.1	44.3	44.0	45.8	40.7	-17.2	0.60
75-79	48.4	46.6	45.3	39.3	49.6	46.0	48.5	43.7	43.1	-11.0	0.35
80-84	52.7	48.1	42.7	51.0	43.3	50.0	54.8	53.8	54.7	3.8	0.18
85+	54.1	54.5	63.2	56.5	68.4	57.9	66.7	65.6	58.0	7.2	0.12
Total	32.4	32.8	32.0	30.2	33.6	31.0	34.4	30.7	33.0	2.0	0.92
Total (age-adjusted to 2016 hemorrhagic stroke patients)	33.3	34.3	32.7	30.9	33.8	31.0	34.6	31.0	33.0	-0.8	0.75

*hemorrhagic stroke includes ICD-10 codes from I61 – I62

†change is defined as = (2016 – 2008/2008) x 100%

‡Mann-Kendall trend test

Table S14. Proportion of 28-day all-cause mortality from hemorrhagic stroke for women between year 2008 and 2016 in Malaysia*

Age groups	2008	2009	2010	2011	2012	2013	2014	2015	2016	†Change from 2008 - 2016	‡Trends (p-value)
0-34	10.7	18.2	15.8	16.0	19.8	17.7	18.0	17.6	21.1	96.7	0.12
35-39	27.8	29.0	32.2	25.8	27.3	30.0	28.4	31.0	27.7	-0.4	0.92
40-44	34.2	25.7	25.3	27.7	16.2	26.6	23.6	27.7	29.0	-15.3	0.92
45-49	31.7	36.8	32.2	29.4	27.9	26.1	30.5	25.6	30.0	-5.7	0.08
50-54	39.7	38.9	34.0	26.8	29.4	34.3	30.8	30.0	37.6	-5.4	0.47
55-59	38.7	37.4	34.6	33.7	31.4	29.6	34.1	31.7	35.3	-8.6	0.18
60-64	40.9	42.1	38.5	36.6	45.0	34.1	39.2	38.1	35.9	-12.2	0.18
65-69	43.5	42.1	39.4	35.4	36.5	37.4	44.5	33.8	36.5	-15.9	0.25
70-74	34.4	41.7	44.4	47.4	36.9	40.5	52.0	42.4	48.0	39.8	0.12
75-79	52.4	50.4	42.6	49.7	51.9	52.3	54.0	42.4	52.3	-0.3	0.75
80-84	59.2	57.8	48.4	52.9	50.0	46.0	54.6	51.6	54.2	-8.5	0.47
85+	62.3	53.1	54.2	51.2	40.0	47.1	58.2	49.5	58.0	-6.9	0.60
Total	37.9	38.3	35.9	35.2	34.2	34.6	38.6	34.2	37.5	-0.9	0.35
Total (age-adjusted to 2016 hemorrhagic stroke patients)	38.1	38.6	35.6	34.8	34.0	34.1	37.6	33.7	37.5	-1.5	0.18

*hemorrhagic stroke includes ICD-10 codes from I61 – I62

†change is defined as = (2016 – 2008/2008) x 100%

‡Mann-Kendall trend test

Table S15. Baseline characteristics of hospital admissions for subarachnoid hemorrhage from 2008 to 2016

	Year																	
	2008 (n)		2009 (n)		2010 (n)		2011 (n)		2012 (n)		2013 (n)		2014 (n)		2015 (n)		2016 (n)	
Hospital admissions for stroke (excluding transfers)		438		450		525		605		399		627		764		698		693
Baseline characteristics	n	%	n	%	n	%	n	%	n	%	n	%	n	%	n	%	n	%
Mean age (SD)	50	(21)	51	(21)	49	(22)	50	(22)	49	(22)	50	(22)	50	(21)	50	(22)	50	(21)
Age groups																		
0-34	91	20.8	86	19.1	124	23.6	130	21.5	90	22.6	145	23.1	162	21.2	155	22.2	149	21.5
35-39	23	5.3	21	4.7	23	4.4	32	5.3	20	5.0	28	4.5	58	7.6	43	6.2	41	5.9
40-44	35	8.0	42	9.3	42	8.0	51	8.4	33	8.3	29	4.6	40	5.2	50	7.2	58	8.4
45-49	45	10.3	51	11.3	45	8.6	50	8.3	38	9.5	60	9.6	66	8.6	48	6.9	57	8.2
50-54	42	9.6	37	8.2	50	9.5	45	7.4	30	7.5	66	10.5	75	9.8	69	9.9	57	8.2
55-59	32	7.3	42	9.3	53	10.1	62	10.2	48	12.0	66	10.5	82	10.7	66	9.5	75	10.8
60-64	55	12.6	43	9.6	54	10.3	63	10.4	37	9.3	56	8.9	57	7.5	85	12.2	60	8.7
65-69	39	8.9	31	6.9	36	6.9	53	8.8	40	10.0	52	8.3	87	11.4	53	7.6	73	10.5
70-74	37	8.4	43	9.6	57	10.9	56	9.3	25	6.3	60	9.6	62	8.1	51	7.3	50	7.2
75-79	16	3.7	32	7.1	21	4.0	31	5.1	14	3.5	36	5.7	46	6.0	42	6.0	47	6.8
80-84	14	3.2	10	2.2	16	3.0	17	2.8	18	4.5	19	3.0	21	2.7	27	3.9	17	2.5
85+	9	2.1	12	2.7	4	0.8	15	2.5	6	1.5	10	1.6	8	1.0	9	1.3	9	1.3
Sex																		
Men	248	56.6	230	51.1	271	51.6	323	53.4	205	51.4	352	56.1	410	53.7	375	53.7	367	53.0
Women	190	43.4	220	48.9	254	48.4	282	46.6	194	48.6	275	43.9	354	46.3	323	46.3	326	47.0

Ethnicity																		
Malay	232	53.0	221	49.1	271	51.6	338	55.9	184	46.1	316	50.4	365	47.8	358	51.3	363	52.4
Chinese	113	25.8	124	27.6	121	23.0	141	23.3	107	26.8	170	27.1	179	23.4	158	22.6	154	22.2
Indian	26	5.9	27	6.0	34	6.5	28	4.6	23	5.8	39	6.2	38	5.0	30	4.3	50	7.2
Others	67	15.3	76	16.9	65	12.4	96	15.9	85	21.3	102	16.3	180	23.6	145	20.8	123	17.7
Unknown	0	0.0	2	0.4	34	6.5	2	0.3	0	0.0	0	0.0	2	0.3	7	1.0	3	0.4
Median length of hospital stay† (IQR)	3	(6)	3	(6)	3	(6)	3	(6)	3	(7)	3	(6)	3	(7)	3	(6)	4	(6)

*subarachnoid hemorrhage includes ICD-10 codes I60 †length of stay includes the duration between hospital transfers

Table S16. Incidence of hospitalized subarachnoid hemorrhage for men between year 2008 and 2016 in Malaysia*

Age groups	2008	2009	2010	2011	2012	2013	2014	2015	2016	†Change from 2008 - 2016	‡Trends (p-value)
0-34	0.7	0.5	0.8	0.9	0.6	1.0	1.0	1.0	1.0	35.5	0.05
35-39	1.6	1.4	1.0	1.7	0.7	1.4	3.0	2.2	2.0	30.9	0.35
40-44	2.0	2.2	2.0	2.9	1.7	1.8	2.4	2.5	2.8	39.8	0.35
45-49	2.5	2.6	2.6	2.8	2.2	3.6	4.3	1.8	2.9	14.4	0.35
50-54	3.2	2.6	2.7	2.3	1.7	4.4	4.1	3.9	2.8	-11.4	0.75
55-59	2.9	2.1	3.7	5.8	3.9	4.5	6.7	4.0	4.5	53.8	0.05
60-64	9.0	4.6	5.5	5.7	2.9	6.0	3.5	7.4	6.1	-31.7	0.75
65-69	6.3	3.8	4.4	8.8	5.0	6.5	11.9	4.4	8.2	31.3	0.35
70-74	6.7	8.9	11.5	9.7	4.8	11.7	11.0	10.6	9.7	44.8	0.60
75-79	5.0	12.6	6.4	7.9	3.2	10.2	11.0	15.4	11.5	131.2	0.12
80-84	10.5	9.9	7.9	12.6	11.0	14.0	11.9	13.8	9.0	-14.4	0.47
85+	5.3	10.3	2.3	7.1	0.0	2.0	7.6	5.3	3.1	-42.7	0.67
Total	1.6	1.4	1.6	2.0	1.2	2.1	2.4	2.2	2.1	30.3	0.05
Total (age-adjusted to 2016 Malaysian population)	1.7	1.5	1.7	2.1	1.3	2.1	2.4	2.2	2.1	20.3	0.12
Total (age adjusted to WHO standard world population)	2.0	1.8	1.9	2.3	1.4	2.4	2.8	2.5	2.1	6.1	0.18

*subarachnoid hemorrhage includes ICD-10 codes I60

†change is defined as = (2016 – 2008/2008) x 100%

‡Mann-Kendall trend test

Table S17. Incidence of hospitalized subarachnoid hemorrhage for women between year 2008 and 2016 in Malaysia*

Age groups	2008	2009	2010	2011	2012	2013	2014	2015	2016	†Change from 2008 - 2016	‡Trends (p-value)
0-34	0.3	0.3	0.4	0.5	0.3	0.5	0.6	0.5	0.4	67.2	0.08
35-39	0.8	0.5	0.9	1.4	1.3	1.1	1.9	1.2	1.3	74.9	0.08
40-44	1.6	2.2	2.4	2.1	1.7	1.3	1.7	2.0	2.6	56.8	0.75
45-49	3.1	3.0	2.3	2.8	1.7	2.9	2.6	3.0	3.1	0.3	0.92
50-54	2.4	2.6	3.6	3.1	2.1	3.3	4.5	3.8	3.5	44.1	0.12
55-59	1.9	5.4	4.0	4.1	3.0	4.4	5.3	4.7	5.5	193.0	0.08
60-64	5.1	3.9	5.2	7.7	4.3	4.6	6.4	6.7	4.4	-12.3	0.60
65-69	7.1	6.2	7.8	7.8	6.3	8.0	10.6	7.3	9.6	34.1	0.12
70-74	9.9	10.9	14.9	13.5	6.5	12.6	12.4	10.2	6.8	-32.0	0.47
75-79	8.5	13.5	8.9	13.2	6.4	13.2	15.5	10.9	15.5	82.1	0.25
80-84	11.0	3.9	13.6	9.9	13.6	12.5	12.2	16.0	11.2	1.5	0.47
85+	13.9	15.7	5.3	19.1	9.6	11.8	5.5	6.5	9.5	-31.2	0.35
Total	1.3	1.5	1.6	1.9	1.2	1.7	2.1	1.9	1.9	49.4	0.05
Total (age-adjusted to 2016 Malaysian population)	1.4	1.6	1.8	2.0	1.3	1.8	2.2	1.9	1.9	32.1	0.12
Total (age-adjusted to WHO standard world population)	1.6	1.8	2.0	2.3	1.5	2.1	2.5	2.2	2.1	29.2	0.12

*subarachnoid hemorrhage includes ICD-10 codes I60

†change is defined as = (2016 – 2008/2008) x 100%

‡Mann-Kendall trend test

Table S18. Proportion of 28-day all-cause mortality from subarachnoid hemorrhage for men between year 2008 and 2016 in Malaysia*

Age groups	2008	2009	2010	2011	2012	2013	2014	2015	2016	†Change from 2008 - 2016	‡Trends (p-value)
0-34	14.5	13.8	12.6	7.0	12.7	8.9	5.0	12.6	11.7	-19.6	0.08
35-39	20.0	18.8	13.3	26.3	28.6	20.0	11.1	13.3	12.5	-37.5	0.25
40-44	33.3	36.4	15.8	23.3	29.4	52.9	20.8	37.9	29.0	-12.9	0.92
45-49	50.0	8.3	37.5	25.0	38.1	24.2	24.4	27.8	18.5	-63.0	0.35
50-54	24.0	30.0	33.3	26.3	40.0	26.8	41.7	40.0	30.8	28.2	0.14
55-59	30.0	25.0	40.0	25.0	27.6	19.4	34.8	21.9	32.4	7.8	0.83
60-64	28.6	27.3	31.0	23.3	53.3	25.8	30.4	29.8	29.4	2.9	0.92
65-69	27.8	41.7	7.7	35.7	50.0	30.4	31.1	38.9	27.3	-1.8	0.92
70-74	13.3	25.0	37.5	20.8	36.4	16.1	33.3	50.0	53.8	303.8	0.08
75-79	40.0	42.9	37.5	54.5	75.0	57.1	55.6	50.0	30.0	-25.0	0.92
80-84	16.7	50.0	80.0	22.2	42.9	77.8	11.1	18.2	14.3	-14.3	0.35
85+	50.0	0.0	100.0	33.3	0.0	100.0	75.0	50.0	50.0	0.0	0.83
Total	25.0	23.9	25.5	21.7	30.7	23.6	24.1	27.5	24.3	-3.0	0.75
Total (age-adjusted to 2016 subarachnoid hemorrhage patients)	25.2	24.6	24.9	22.1	33.0	25.2	23.5	27.7	24.3	-3.8	0.35

*subarachnoid hemorrhage includes ICD-10 codes I60

†change is defined as = (2016 – 2008/2008) x 100%

‡Mann-Kendall trend test

Table S19. Proportion of 28-day all-cause mortality from subarachnoid hemorrhage for women between year 2008 and 2016 in Malaysia*

Age groups	2008	2009	2010	2011	2012	2013	2014	2015	2016	†Change from 2008 - 2016	‡Trends (p-value)
0-34	22.7	10.7	13.5	15.9	11.1	9.1	8.2	11.5	19.6	-13.9	0.60
35-39	25.0	40.0	50.0	7.7	38.5	15.4	31.8	30.8	17.6	-29.4	0.47
40-44	47.1	25.0	21.7	28.6	18.8	16.7	6.3	28.6	25.9	-44.9	0.29
45-49	20.0	25.9	38.1	27.3	47.1	22.2	20.0	20.0	20.0	0.0	0.32
50-54	52.9	58.8	20.7	30.8	26.7	28.0	28.2	32.4	25.8	-51.3	0.47
55-59	8.3	30.0	17.9	50.0	42.1	23.3	41.7	38.2	36.6	339.0	0.47
60-64	30.0	33.3	52.0	54.5	31.8	44.0	23.5	18.4	26.9	-10.3	0.25
65-69	47.6	36.8	39.1	40.0	45.8	34.5	31.0	25.7	40.0	-16.0	0.21
70-74	54.5	43.5	42.4	43.8	42.9	31.0	40.0	44.4	50.0	-8.3	0.92
75-79	63.6	77.8	46.2	70.0	50.0	54.5	32.1	55.6	37.0	-41.8	0.18
80-84	62.5	25.0	81.8	62.5	54.5	30.0	75.0	43.8	20.0	-68.0	0.29
85+	71.4	75.0	0.0	58.3	100.0	55.6	50.0	80.0	57.1	-20.0	0.92
Total	39.5	36.8	33.1	38.7	37.1	28.4	28.0	29.4	30.4	-23.1	0.08
Total (age-adjusted to 2016 subarachnoid hemorrhage patients)	37.3	36.6	32.4	38.2	36.6	27.8	27.8	30.9	30.4	-18.6	0.47

*subarachnoid hemorrhage includes ICD-10 codes I60

†change is defined as = (2016 – 2008/2008) x 100%

‡Mann-Kendall trend test
